# Assessing the Effects of a Tobacco Tax Reform on the Industry Price-Setting Strategy

**DOI:** 10.3390/ijerph181910376

**Published:** 2021-10-02

**Authors:** Jose Angelo Divino, Philipp Ehrl, Osvaldo Candido, Marcos Aurelio Pereira Valadao

**Affiliations:** 1 Graduate Program of Economics, Catholic University of Brasília, Brasília 71966-700, Brazil; philipp.ehrl@p.ucb.br (P.E.); osvaldoc@p.ucb.br (O.C.); 2 School of Public Policy and Government, Getulio Vargas Foundation, Brasilia 70830-051, Brazil; profvaladao@yahoo.com.br

**Keywords:** Bill no. 3887-2020, tobacco tax, tax reform, cigarette prices, cigarette consumption

## Abstract

In July 2020, the Executive Power submitted Bill no. 3887-2020 as the first step towards a wide reform of the Brazilian tax system. It will replace the current PIS/COFINS (charged on turnover of companies) by the CBS (a tax on goods and services), which includes a special regime for cigarettes. The novelty is that the specific cigarette tax will be charged on the highest retail price per cigarette brand across the country. This research simulates three scenarios that differ according to the price-setting strategy of the tobacco industry in reaction to the proposed tax reform. In all simulations, the tax reform would result in considerably higher cigarette prices, lower cigarette consumption, higher tax collection, and an implicit minimum price that is far above the current official price floor. Furthermore, the price dispersion and cross-border shopping across states would be reduced because prices and tax burden per brand would tend to be the same across the country due to the dominant price-setting strategy in the cigarette industry.

## 1. Introduction

Over the past few decades, Brazil has significantly reduced the prevalence of smoking, from 34.8 percent in 1989 to approximately 10.5 percent in Brazilian state capitals in 2019 [1,2]. This outstanding decrease can be attributed to the implementation of strong tobacco control policies, which include smoking restrictions, advertising regulations, cutting economic incentives to tobacco farming and, above all, increasing taxes on tobacco products [3,4]. According to the international evidence, increasing taxes on tobacco products is the most effective public policy to reduce smoking; while at the same time, increased tax revenue can be used to cover the costs of treatment for tobacco-related diseases by the public health system [5,6,7,8].

While there are currently two Constitutional Amendment Bills in the National Congress that could result in a change in the tax system at both federal and state/local levels, the Executive Power has also been working on a separate tax reform proposal and has submitted a bill (no. 3887-2020) to the Congress. According to the most recent publicly available information as of august 2021, the latter has the highest chances of being implemented. Bill no. 3887-2020 is supposed to replace the current PIS/COFINS (a federal social contribution levied on turnover of companies, with a special tax regime for cigarettes) with the CBS (Social Contribution on Operations with Goods and Services). Designed as a social contribution, the CBS is a general, non-cumulative tax on consumption. Under this bill, there would be no change in the IPI (a federal consumption tax on manufactured products) and ICMS (a subnational consumption tax), which are other taxes levied on cigarettes. The CBS includes a special regime for cigarettes: a 22 percent ad valorem tax rate on the highest price per brand plus a specific tax of BRL 1.10 per pack. This reform would yield a substantially higher tax burden for the new CBS compared to the current PIS/COFINS that it would replace. The combination of an ad valorem and a fixed component, however, is already part of the current cigarette tax regime.

The main distinction compared to the current system is that the reform would harmonize tax rates that are nowadays quite varied across the Brazilian states. As stated in Bill no. 3887-2020, if a cigarette brand is sold at different prices across states, the tax base will be the highest retail price in any state, regardless of the quantity traded. For instance, if retail prices for a given brand in states A, B, C, and D are BRL 10.00, in states E, F, G, H, and J are equal to BRL 12.50, and in state K the is BRL 14.00, then the whole production (sold in states A to K) will be taxed at BRL 14.00 × 22% + 1.10 per pack. Therefore, the total CBS would be BRL 4.18 per pack across all states.

The present paper investigates how this tax reform might affect the cigarette industry’s price setting strategy and analyzes the potential impacts of Bill no. 3887-2020 on cigarette prices, smoking prevalence, tax collection, and cigarette consumption considering effects at the federal and state levels. Assuming a successful implementation of the new CBS through Bill no. 3887-2020, this research simulates three price-setting scenarios, where the tobacco industry adjusts prices to: (a) match the highest price per brand in all the states (Scenario I); (b) keep the markup (margin) per cigarette brand and state at the average pre-tax reform level (Scenario II); (c) keep the markup per cigarette brand and state at the highest pre-tax reform level (Scenario III). This paper uses the latest available data on smoking behavior from a nationally representative survey repeated in 2018 and 2019 as the baseline scenario for the simulations [2,9].

The cigarette market is divided into four price categories (PC). All the brands sold below the universally binding minimum legal price are considered illicit brands [5,10,11] and listed as price category 1 (PC1). The legal cigarette market is divided into low price brands (PC2), medium price brands (PC3), and high price (or premium) brands (PC4). In order to incorporate the smokers’ sensitivities to price changes as precisely as possible, this research estimates a combination of conditional and unconditional price-elasticities by geographical region and PC. Working with four price categories thus generates price heterogeneity in the simulations while keeping clarity of other elements such as the price elasticity per PC.

The tobacco industry will most likely react to the higher after-reform tax burden by increasing retail prices such that it adjusts the markup over the costs incurred from production to the point of sale. In the current situation, markups differ across states because the tax burden and logistics costs vary while production costs are basically the same. In all scenarios, the tax reform proposed by Bill no. 3887-2020 would result in higher cigarette prices and lower cigarette consumption. The tax burden would increase relative to the current situation. Since the tax base is defined by the highest price per brand across the country, producers would lose the incentive to charge different prices across states. Another relevant consequence of the reform is that cigarette prices per brand would tend towards uniformity across states. This induced behavior by the tax reform would reduce both the cigarette price gap and cross-border shopping across states.

Moreover, the simulations imply that there will be an implicit price floor below which cigarette sales are not feasible by the industry. In the most likely scenario (I), this price is 8.40 BRL, which is well above the current official minimum price of 5.00 BRL per pack.

These findings complement the literature on the effects of tobacco tax changes, primarily those rare cases that address a harmonization of prices. Ballester et al. also conclude that the variation of cigarette prices across US states weakens the effectiveness of policy measures that intend to reduce smoking [12]. Scollo et al. report positive effects from Australia’s experience, where the opportunity for cross border tax evasion was eliminated in 1997 [13]. The simulations by López-Nicolás and Branston stress that, even for the European Union, an elimination of cross-country cigarette price differences would be beneficial [14]. Similarly, Freitas-Lemos et al. found that reducing price differences between tobacco products is effective to reduce demand [8]. The authors recommend designing taxes for all tobacco related products in proportion to product risk, such that less harmless medicinal nicotine would incur lower taxes than industrialized or roll-your-own tobacco. This interesting topic could be considered in the current tax reform debate in order to strengthen Brazilian tobacco control measures, because some consumers will tend to avoid spending more by changing from industrialized cigarettes to other tobacco products [15,16].

The positive effects of higher cigarette taxes and lower cigarette consumption are widely recognized and seem to apply to high-, middle-, and low-income countries [5,8,17,18,19]. The current study also supports the notion that mixed excise systems lead to good outcomes because prices as well as tax revenue increase [20,21]. The specific value tax decreases the cigarette price gap and thus mitigates the switching demand effect to cheaper brands after a tax increase. However, the choice between specific and ad valorem tax (or both) also depends on the cigarette market structure (monopoly, oligopoly, or competition) [22]. Despite the benefits of tax increases, some authors highlight that there are political and technical challenges involved [23]. Simulations for Vietnam, for instance, show that tax increases under a pure ad valorem system have modest impacts on the smoking rate and thus do not deliver the desired results [24]. China has also recently changed its cigarette tax structure to a mixed system [25], but it lacks a minimum price and higher tax burden in order to achieve a further reduction of tobacco consumption [26,27].

In sum, the partial tax reform proposed under Bill no. 3887-2020 is a step forward for tobacco control in Brazil as it would significantly reduce cigarette consumption while still generating additional tax revenue. The extra resources could be either earmarked to social programs and health expenses or used freely by the government to support the public health system and deter people from smoking.

## 2. Materials and Methods

### 2.1. Data and Sources

The primary source of information on smoking behavior in Brazil is Vigitel—an annual national survey of the Ministry of Health conducted by phone calls to individuals randomly chosen in the 26 state capitals and the Federal District [2,9]. By applying appropriate sample weights, the information in this data set becomes representative for the entire population. The purpose of Vigitel is surveillance of risk and protective factors for chronic diseases.

To increase the precision of the estimates, data from the two most recent years—2018 and 2019—is pooled. According to Vigitel, the average share of smokers varies between 4 and 13 percent across the Brazilian states [2,9]. Altogether, 5314 smokers are observed with complete information about their usual consumption and the price of cigarettes in their last purchase.

**Figure 1:**
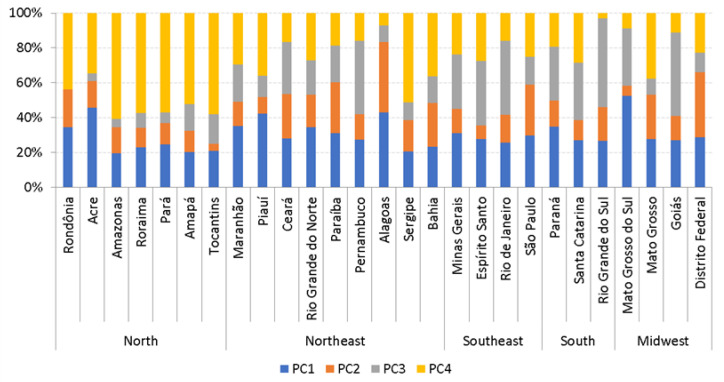
Distribution of smokers by price categories across states and regions.

The cigarette market is divided into four different price segments. Price Category 1 (PC1) represents cigarettes that were purchased at a price below the official minimum price. Thus, these brands are classified as illicit (or illegal), in line with Divino et al. [5]. The remainder of the market is divided into low, medium, and high price categories, according to the percentiles 33 and 66. That is, the legal market is split into three equally large segments. Figure 1 presents the distribution of smokers by price categories, states, and geographical regions.

Although representative, the low number of smokers in Vigitel is critical for the estimation of price-elasticities by states and price categories. Therefore, two other representative individual surveys, the National Household Sample Survey (PNAD) of 2008 [28] and the National Health Survey (PNS) of 2013 [29] are used. Both of them contain the number of cigarettes an individual smoked per day and how much was paid for the cigarettes in the last purchase. Individual socio-economic characteristics—such as gender, income, years of smoking—are used as control variables in the price-elasticity estimation, as will be explained in the next section. Further information about the dataset and descriptive statistics can be found in Divino et al. [5]. As a robustness check, the elasticity estimation with Vigitel data [2,9] yielded similar results but larger confidence intervals.

The exact number of inhabitants per state from the IBGE (Brazilian Institute of Geography and Statistics) [30] is multiplied by the consumption patterns and the current share of smokers from Vigitel [2] to derive the aggregate cigarette consumption. Finally, average cigarette prices are updated by the aggregate, wide consumer price index for the tobacco sub-category (IPCA-Tobacco) in the same period and for each state. Because this information is not available for all states, regional averages are used as a substitute when needed. Further information on the current tax system, officially registered cigarette retail prices, and cigarette tax revenue were obtained from Receita Federal [31].

### 2.2. Methodology

#### 2.2.1. Price Elasticity Estimation

A price elasticity of consumption is a measure that indicates how many percentage points cigarette consumption would change if cigarette prices changed by one percent. Based on the procedure described in Divino et al. [5], the price elasticity is obtained in two steps. The first step provides an estimate of how many individuals would quit or start smoking due to higher or lower cigarette prices using a probit model. The result of the second estimation indicates how continuing smokers would adjust the intensity of their current consumption after a price change. The latter conditional price elasticities are estimated by a linear regression from the log of cigarette consumption on the log of cigarette price interacted with price category and regional indicators, controlling for differences in age, education, years of smoking, income, and gender among individuals.

The combination of these so-called prevalence and conditional elasticities yields the total price elasticity that is used in the simulations. Note that the price elasticities are specific for each geographic region and price category. In both estimations, the individually reported price is substituted by the state average price to avoid the endogeneity bias that occurs, because consumers may adjust to price changes by switching to a cheaper brand. According to the meta-analysis studies, the methodological choice of our price elasticity estimations corresponds to those most frequently used in other studies and these choices tend to generate elasticities that are well in the middle of the price elasticity range found in Gallet & List [32].

#### 2.2.2. Tax Reform Simulations

The simulated scenarios depart from the current tax structure on cigarettes, which is then changed to a new tax scheme defined by the Bill no. 3887-2020 with the introduction of the new CBS, replacing the former PIS/COFINS. Three alternative scenarios for the industry price-setting strategy in response to the new CBS are considered.

#### 2.2.3. Baseline Scenario

The total tax collection from tobacco related products in 2019 was about 17.75 BRL billion. Since Receita Federal does not publish tax revenues at subnational levels, the model is calibrated in the baseline scenario to match this aggregate tobacco tax collection. The adjustment parameter in the calibration is the size of the illicit market. Reference [10] compared four different methods to estimate the size of the illicit cigarette market in five Brazilian cities and conclude that while personal interviews, household garbage analysis and litter collection yield comparable estimates, the Vigitel phone interviews suggest a lower extension. We proportionally increase the extension of PC1, under the restriction that the share of smokers in PC2, PC3, and PC4 does not become negative, until the simulated tobacco tax revenue reaches the observed value in 2019. An average value of 30 percentage points is obtained. That is, each of the price categories in the legal market is reduced by the same percentage, which is then added to the market share of illegal cigarettes. Note that current tax rules are used to calculate the monthly tobacco tax collection per state for the IPI, PIS/CONFINS, and ICMS. In the absence of further information about the brand of cigarettes purchased, the Special Rule for IPI calculation is considered throughout the simulations. The ICMS tax rates on tobacco products for each Brazilian State are obtained from Ribeiro and Pinto. [33]

As can be seen from Figure 1, the share of the illicit market varies between 53% in Mato Grosso do Sul, a state in the Midwest region bordering Paraguay, and 19% in the Amazonas state in the North region. These numbers are now well in line with those obtained by Szklo et al. [10].

An explicit assumption in the reform scenario simulations is that, once the size of the illegal market is adjusted in the baseline scenario, it remains constant. In other words, we conjecture that individuals do not switch from the legal to the illegal market after the tax reform. This behavior is rational and may occur in many instances because the price in the illicit market closely follows the price of legal cigarettes. [11] A second justification for our assumption is that, under a committed tax administration, cigarette price increases are not followed by a growing illicit market [34,35]. Finally, the lack of credible cross-price elasticity estimations justifies this simplification more than assuming any other arbitrary behavior.

In the following three scenarios, the key variable that drives the outcomes is the cigarette producers’ markup over (constant) production costs. This markup then determines, in combination with the exogenous tax reform, the retail prices of cigarettes in the different price categories and across states. Once prices are determined, we apply price elasticities to derive the cigarette consumption and tax collection in each simulated scenario.

#### 2.2.4. Scenario I—Minimum Price Adjustment

As a response to the tax reform, a no price-adjustment strategy by the tobacco industry is not feasible because this would imply negative profits for some cigarette brands that would have a tax burden above 100 percent of the retail price, depending on the state. The industry would most likely react to the new tax structure by increasing retail prices such that profits are positive again for all price categories. This is possible by choosing the highest price per brand so that tax burden is smaller than 100 percent for all price categories. This scenario represents a minimum price adjustment by the cigarette industry to keep positive profits after the tax reform resulting from the Bill no. 3887-2020.

Because the proposed reform specifies that the tax incidence on cigarettes will be based on the highest retail price per brand in the country, this research assumes that cigarette producers would rationally charge the same price for a given brand across all states. This assumption is maintained in this and in the following scenarios due to specific features of Bill no. 3887-2020 are discussed in Section 2. The rationale for this is that if a producer sets price below the highest price in the country for a given brand, then its tax burden would increase, and consequently, the markup would reduce for that brand. Under this reform, the producer would pay the same amount of tax for both the lower and the higher retail price. Thus, there would be no reason to sell below the highest retail price per brand across states.

Since the price-adjustment rule in this scenario implies choosing the lowest price that maintains the tax burden strictly below 100 percent (given that it does not make sense to have the retail price below the amount of tax due), the producers would not transfer the tax burden increase to the retail prices in full. That is, if cigarette production and logistics costs do not change, the producers would be implicitly accepting a reduction in their markup (profit margin).

Smokers adjust their consumption behavior according to the estimated total price-elasticity of demand. It is important to note that, by assumption, the distribution of consumers by price category does not change. They do not switch price categories, but instead either adjust the intensity of their cigarette consumption or quit smoking.

#### 2.2.5. Scenario II—Average Pre-Reform Markup Price-Adjustment

Scenario II allows the cigarette producers to choose any price which leads to a markup above the one implicitly defined in Scenario I.

In Scenario II, the assumption of highest price-setting is kept and additionally, producers choose to maintain the average-weighted markup of the baseline scenario. This means that, for some brands and states, producers might transfer only part of the tax burden increase to the retail prices to keep the pre-reform average markup. In some states, the markup may increase, while in others it may decrease up to the average level. The average-weighted values are obtained considering the share of consumers across Brazilian states.

This research assumes that the cigarette producers not only adjust their prices to avoid losses, but also adjust the markup over the production cost from production to point of sale. In the current tax structure, markups differ across states because the tax burden and logistics costs vary across the states, while production costs are basically the same. In this second scenario, the markup is set to its current average value across all states. Consequentially, cigarette prices as well as profits are higher than under Scenario I.

#### 2.2.6. Scenario III—Maximum Pre-Reform Markup Price-Adjustment

Scenario III is an extreme scenario. In addition to the highest price-setting assumption, producers do not accept any reduction to their markup. Therefore, they keep the highest retail price combined with the highest markup among the Brazilian states resulting from the new tax burden.

This scenario is much like the previous one except that the cigarette industry adjusts its price-setting strategy to preserve markups (from production to point of sale). Under the new tax structure, the industry would have an incentive to choose the highest price per brand that at least maintains the pre-reform markup across states.

## 3. Results and Discussion

### 3.1. Price Elasticity of Cigarette Consumption

Table 1 reports the estimated price elasticities by geographic region and price category. The prevalence component indicates that a price increase of 10 percent would reduce smoking prevalence by about two percent. The other component of the total price elasticity indicates how much smokers who continue to smoke reduce their consumption of cigarettes.

The differences between the total elasticities show that richer regions tend to be less sensitive to price increases. Moreover, individuals who buy brands that are more expensive respond less to price changes. Thus, the total elasticity estimates indicate that low price brands (PC1 and PC2) sold in the poorest regions of the country (northeast and north) present the highest sensitivities to price changes in cigarettes. On the other hand, consumers of high-price brands in the wealthier southern region are the least price sensitive, according to common expectations. Across all Brazilian states, a 10 percent price increase would decrease consumption between 3.9 percent for the high-price cigarettes in the south and 8.6 percent for illegal cigarette consumption in the northeast.

### 3.2. Tax Reform Simulations

The simulated scenarios depart from the current tax structure on cigarettes, which is changed to accommodate the new tax scheme defined by the Bill no. 3887-2020 with the introduction of the new CBS, replacing the former PIS/COFINS. Three alternative scenarios for the industry price-setting strategy in response to the new CBS are considered.

Notice that tobacco tax revenue refers to cigarette tax collection attributable to a given state and not that the revenue is available to this state. It corresponds to the total cigarette tax revenue accrued in a specific state (or states) as the sum of federal (CBS and IPI) and state (ICMS) taxes on cigarette collected within each state territorial limits.

### 3.3. Scenario I—Minimum Price Adjustment

The industry will most likely react to the new tax structure by increasing retail prices such that profits are positive for all price categories. According to our simulations, this price would be at least 8.40 BRL. After this adjustment, the low- and medium-price categories essentially collapse to the same price. Moreover, this scenario implies that there will be an implicit floor price below which cigarette sales are not feasible by the industry because profits would be negative. This price (8.40 BRL) is well above the current official floor price of 5.00 BRL per cigarette pack.

Since the tax base is the highest price per brand across the country, producers will lose the incentive to charge different prices across states. That is, another relevant consequence of the reform is that cigarette prices per brand would become uniform across states. This induced behavior by the tax reform will reduce both cigarette price gap and cross-border shopping across states.

The simulations of Scenario I indicate that the tax reform would raise cigarette tax revenue by 30.7 percent, or 5.4 billion BRL per year relative to the baseline. High-price cigarettes would be 15.20 BRL per pack. These price changes correspond to an increase of 56.3, 6.3, and 19.8 percent for price categories 2 to 4, respectively, compared to the baseline. In turn, cigarette consumption would decrease by 38.1, 3.2, and 9.6 percent for categories 2 to 4, respectively.

### 3.4. Scenario II—Average Pre-Reform Markup Price-Adjustment

In the second scenario, we assume that the cigarette producers not only adjust their prices to avoid losses but also change the markup over the production cost from production to the retail point. In this scenario, the markup is set to its current average value across all states. Consequentially, cigarette prices as well as profits are higher than under Scenario I.

As a result of the average pre-reform markup, prices for categories 2 to 4 will be equal to 10.0, 13.1, and 19.4 BRL, respectively. Since consumers are price sensitive, cigarette consumption falls by 58, 33, and 25 percent, respectively, while the tax burden will be between 87 percent (PC2) and 74 percent (PC4). These numbers indicate that the aggregate tax collection will be lower than under Scenario I, but still 23.5 percent higher than the baseline tax collection.

### 3.5. Scenario III—Maximum Pre-Reform Markup Price-Adjustment

Under this scenario, the cigarette industry adjusts its price-setting strategy to preserve markups (from production to the retail point). Under the new tax structure, the industry has an incentive to choose the highest price per brand that at least keeps the pre-reform markup across states. In this case, the new tax burdens for PCs 2–4 are 84.9, 78.9, and 72.5 percent, respectively. The single prices per cigarette pack and brand for PCs 2 to 4 are 11.06, 14.87, and 23.38 BRL, respectively. Under this scenario, cigarette consumption decreases sharply in PCs 2 to 4 by 71.5, 44.6, and 40.5 percent, respectively. Because of this substantial decrease in smoking, tax collection increases only by 15.7 percent per year relative to the baseline scenario. Cross-border shopping across states also reduces because prices per brand will tend to be the same across the country. The three simulated scenarios altogether are summarized in Table 2 below.

## 4. Conclusions

This paper considers the tobacco section of Bill no. 3887-2020 and analyzes the potential impacts of the tax reform on cigarette prices, cigarette consumption, and tax collection at both the federal and state levels. This research simulates alternative price responses of the cigarette industry to the new tax scheme and evaluates potential impacts of these responses on the cigarette market and tax collection.

One of the main findings is that, no matter how the tobacco industry responds to the tax increase, Bill no. 3887-2020 would increase cigarette taxes and prices, resulting in a decrease in tobacco consumption. Despite this decrease in consumption of cigarettes in the population, the change in tax revenue is still positive. The tax reform would reduce the gap between low and high price cigarettes as cheaper cigarettes’ prices would increase more than premium brands, something that is of major political relevance.

Moreover, the tax reform proposed under Bill no. 3887-2020 would result in an implicit minimum price that is far above the current official floor price. In all simulated scenarios, the tax burden—that is, the total tax share in the retail price—increases relative to the current baseline situation and it tends toward uniformity across all states. Consequently, cross-border shopping and the price gap between cigarette brands would be reduced.

Brazilian tax administration is effective in controlling cigarette production and distribution to tackle illicit market through a monitoring system named Control and Tracking System for Cigarette Production (Scorpios) (For more detalied information, see https://www.gov.br/pt-br/servicos/consultar-sistema-de-controle-de-producao-de-cigarros (in Portuguese) (accessed on 19 November 2020)). It comprises a production counter equipment for the control, recording, transmission, and tracking of products throughout the national territory to identify the origin and suppress illegal production and importation, as well as the sale of counterfeit products. All the information is transmitted to the tax administration authority. The fight against cigarette smuggling currently depends heavily on the implementation of the Protocol to Eliminate Illicit Trade in Tobacco Products under the WHO Framework Convention on Tobacco Control.

Because cigarette prices and the tax burden across states are currently different, the distribution of gains from the reform across states is uneven. Yet no Brazilian state experiences tax revenue losses under either scenario, on aggregate. However, the assumption of the stability of the illicit market should be noted here. Thus, a crucial requirement to reap the positive aspects of the tobacco tax reform is curbing illicit trade through consistent and continuous public policies to fight cigarette smuggling.

It is also important to stress that the proposed CBS tax reform increases the retail price of low-price cigarettes relatively more than medium and high price brands. This is desirable under a tobacco control policy perspective because it tends to reduce smoking proportionately more among lower income individuals, who are most likely to buy lower price brands. This finding, however, does not necessarily imply that the tax reform is either progressive or regressive, as the simulations have not analyzed income levels but cigarette price categories instead. Only under the (strong) assumption that low-income groups consume cheaper brands, these individuals would pay relatively more taxes than higher income smokers would by choosing premium brands after the tax increase. This would make the tax reform more regressive. However, this is not necessarily the case because the simulations have addressed only cigarette price categories and not individual income levels.

The CBS implementation, however, is challenging due to the new format of charging a tax rate on the highest price per cigarette brand. In particular, there are potential challenges about computing and using the highest nationwide price per cigarette brand as a tax base, avoiding tax evasion through under-declaration of prices by the industry, and selling cigarettes above the reported price. Thus, coupled with the innovative tax scheme proposed by Bill no. 3887-2020, there must be a strong tax administration to avoid potential tax revenue leakages.

Finally, the analysis has some limitations that are worth mentioning. The simulations follow a partial equilibrium approach, which does not account for neither dynamics over time nor second order effects of price adjustments. These effects would appear in a more complex modelling strategy, such as a dynamic stochastic general equilibrium (DSGE) model that is beyond the scope of the paper. A caveat here is that the share of the tobacco segment in the total output is negligible when compared to other sectors of the economy. It might also be desirable to model the tobacco industry price setting strategy in a richer environment of the production sector and to make policy recommendations about alternative scenarios for the tobacco tax reform under discussion in the Brazilian economy. Some of these improvements are the objectives of our ongoing research, while others are left as suggestions for future research.

## Figures and Tables

**Table 1:** Price-elasticities by regions and price categories.

Region	Prevalence	Total			
		PC1	PC2	PC3	PC4
Northeast	−0.26	−0.86	−0.68	−0.62	−0.57
North	−0.24	−0.73	−0.68	−0.50	−0.48
Southeast	−0.24	−0.56	−0.68	−0.46	−0.42
South	−0.21	−0.51	−0.66	−0.40	−0.39
Midwest	−0.23	−0.69	−0.67	−0.42	−0.47

Notes: PC1 = price category 1 or illicit market, PC2 = low price brands, PC3 = medium price brands, and PC4 = high price brands. Standard errors in our elasticity estimations are robust to heterogeneity. According to these standard errors, the prevalence elasticity is significant at the 10% level, whereas the conditional elasticities are significant at the 1% level.

**Table 2:** Tax reform simulation results across different scenarios.

Feature	Baseline	Scenario I	Scenario II	Scenario III
Tax collection (BRL Bi per year)	17.75	23.20	21.92	20.53
Change (Baseline ref)	---	30.7%	23.5%	15.7%
PC2: Low price brands (BRL)	5.38	8.40	10.03	11.06
Tax burden	78.3%	92.3%	87.3%	84.9%
Share in tax collection	24.06%	21.89%	19.63%	18.1%
Consumption (% change)	---	−38.1%	−58.6%	−71.50%
PC3: Medium price (BRL)	7.90	8.40	13.15	14.87
Tax burden	69.4%	92.3%	81.2%	78.9%
Share in tax collection	35.75%	37.28%	37.56%	38.21%
Consumption (% change)	---	−3.2%	−33.5%	−44.6%
PC4: Premium brands (BRL)	12.84	15.23	19.42	23.38
Tax burden	62.2%	78.5%	74.8%	72.5%
Share in tax collection	40.19%	40.83%	42.81%	43.7%
Consumption (% change)	---	−9.6%	−25.5%	−40.50%

Notes: Scenario I is the minimum price adjustment case, Scenario II defines that the industry implements average pre-reform markup price adjustment, and Scenario III considers the maximum pre-reform markup in each state and price class. The share in total tax collection refers to the percentage of revenue obtained by each price category relative to the total cigarette tax revenue.

## Data Availability

The public data used in the paper are: (1) National Household Sample Survey (PNAD) de 2008, available at https://www.ibge.gov.br/estatisticas/sociais/educacao/9127-pesquisa-nacional-por-amostra-de-domicilios.html?edicao=9128&t=downloads (accessed on 19 November 2020) or at https://ftp.ibge.gov.br/Trabalho_e_Rendimento/Pesquisa_Nacional_por_Amostra_de_Domicilios_anual/2008/Documentacao_pnad2008/Dados.zip (accessed on 19 November 2020); (2) National Health Survey (PNS) de 2013, available at https://www.ibge.gov.br/estatisticas/sociais/saude/9160-pesquisa-nacional-de-saude.html?=&t=downloads (accessed on 19 November 2020) or at https://ftp.ibge.gov.br/PNS/2013/Microdados/Dados/PNS_2013.zip (accessed on 19 November 2020); (3) Risk Factor Surveillance and Protection for Chronic Diseases by Telephone Survey (VIGITEL), 2018, 2019, which is performed annually, available at http://svs.aids.gov.br/download/Vigitel/ (accessed on 19 November 2020), for the years 2018 and 2019 used in the text see at https://portalarquivos2.saude.gov.br/images/pdf/2019/julho/25/vigitel-brasil-2018.pdf (accessed on 19 November 2020); and https://bvsms.saude.gov.br/bvs/publicacoes/vigitel_brasil_2019_vigilancia_fatores_risco.pdf (accessed on 19 November 2020); (4) Cigarette tax revenue at a national level, Federal Revenue Service (Brazil), available at https://www.gov.br/receitafederal/pt-br (accessed on 19 November 2020); (5) Population per state—Brazilian Institute of Geography and Statistics—IBGE—available at https://www.ibge.gov.br/estatisticas/sociais/populacao.html (accessed on 19 November 2020).
